# An Unusual U2AF2 Inhibits Splicing and Attenuates the Virulence of the Human Protozoan Parasite *Entamoeba histolytica*

**DOI:** 10.3389/fcimb.2022.888428

**Published:** 2022-06-17

**Authors:** Gretter González-Blanco, Guillermina García-Rivera, Patricia Talmás-Rohana, Ester Orozco, José Manuel Galindo-Rosales, Cristina Vélez, Odila Salucedo-Cárdenas, Elisa Azuara-Liceaga, Mario Alberto Rodríguez-Rodríguez, Tomoyoshi Nozaki, Jesús Valdés

**Affiliations:** ^1^ Departamento de Bioquímica, Centro de Investigación y de Estudios Avanzados del Instituto Politécnico Nacional (CINVESTAV), CDMX, Mexico; ^2^ Departamento de Infectómica y Patogénesis Molecular, Centro de Investigación y de Estudios Avanzados del Instituto Politécnico Nacional (CINVESTAV), CDMX, Mexico; ^3^ Departamento de Biología Celular, Centro de Investigación y de Estudios Avanzados del Instituto Politécnico Nacional (CINVESTAV), CDMX, Mexico; ^4^ Departamento de Histología, Facultad de Medicina, Universidad Autónoma de Nuevo León, Monterrey, Mexico; ^5^ Posgrado en Ciencias Genómicas, Universidad Autónoma de la Ciudad de México, CDMX, Mexico; ^6^ Laboratory of Biomedical Chemistry, Department of International Health, Graduate School of Medicine, The University of Tokyo, Tokyo, Japan

**Keywords:** intron retention, KH-QUA2 motifs, splicing complex E to A transition, protein–RNA binding, protein–protein interactions

## Abstract

*E. histolytica* is the etiological agent of intestinal amebiasis and liver abscesses, which still poses public health threat globally. Metronidazole is the drug of choice against amebiasis. However, metronidazole-resistant amoebic clinical isolates and strains have been reported recently, challenging the efforts for amebiasis eradication. In search of alternative treatments, *E. histolytica* transcriptomes have shown the association of genes involved in RNA metabolism with the virulence of the parasite. Among the upregulated genes in amoebic liver abscesses are the splicing factors EhU2AF2 and a paralog of EhSF3B1. For this reason and because EhU2AF2 contains unusual KH-QUA2 (84KQ) motifs in its lengthened C-terminus domain, here we investigated how the role of EhU2AF2 in pre-mRNA processing impacts the virulence of the parasite. We found that 84KQ is involved in splicing inhibition/intron retention of several virulence and non-virulence-related genes. The 84KQ domain interacts with the same domain of the constitutive splicing factor SF1 (SF1KQ), both in solution and when SF1KQ is bound to branchpoint signal RNA probes. The 84KQ–SF1KQ interaction prevents splicing complex E to A transition, thus inhibiting splicing. Surprisingly, the deletion of the 84KQ domain in EhU2AF2 amoeba transformants increased splicing and enhanced the *in vitro* and *in vivo* virulence phenotypes. We conclude that the interaction of the 84KQ and SF1KQ domains, probably involving additional factors, tunes down Entamoeba virulence by favoring intron retention.

## 1 Introduction

The splicing of the pre-mRNA takes place in the spliceosome, which is the dynamic enzyme formed by small nuclear ribonucleoproteins (snRNPs) U1, U2, and U4/U6•U5 and multiple auxiliary factors sequentially recruited on the pre-mRNA (Wahl et al., 2009). For an efficient splicing process, broadly conserved *cis* sequences are recognized on the pre-mRNA, the 5′ and 3′ splice sites (ss), a branching site (BS), followed by a polypyrimidine (Py) tract. U1 snRNP binds to the 5′ss, while U2 snRNP recognizes a 3′ss through base recognition with the BS (Shao et al., 2014). Because BS is degenerated in higher eukaryotic cells, the addition of U2 snRNP requires multiple auxiliary factors, including splicing factor 1 (SF1) and the heterodimer U2AF1/2, which consists of two subunits of 35 and 65 kDa, respectively. U2AF2 binds to the Py tract immediately downstream of the BS, and U2AF1 recognizes the 3′ss dinucleotide AG, forming splicing complex A (Wahl et al., 2009). The amino-terminal domain of U2AF2 is enriched in arginine and serine (RS) residues that promote and stabilize the interaction of the U2 snRNP with the BS. The SR-rich domain is followed by a proline-rich domain and three RNA-binding domains, two of which are canonical RNA recognition motifs (RRM) that bind directly to the Py tract of the pre-mRNA adjacent to the 3′ss (Sickmier et al., 2006). The third RRM domain (RRM3) is a protein–protein interaction domain, defined as a U2AF homology motif (UHM) that interacts with SF1 at or near its BS recognition motif (Wu and Fu, 2015). The dual interaction of U2AF2 with Pol II and core components of the U2 snRNP (David et al., 2011) facilitate recruiting of U2 snRNP at the BS, replacing SF1 with the U2 snRNA forming a U2 snRNA/BS hybrid (Valcarcel et al., 1996), thus activating the 3′ss and positioning the A residue of the BS to perform the nucleophilic attack at the 5′ss.

*Entamoeba histolytica* is the protozoan parasite that causes intestinal amebiasis, a public health problem in many developing countries, causing up to 100,000 fatalities per year (Saidin et al., 2019). This parasite is a category B priority biodefense pathogen by the National Institute of Allergy and Infectious Diseases due to its low infectious dose, resistance to chlorine, and environmental stability, properties that can represent a threat that is easily spread through contamination of food and water supplies (Shirley et al., 2018). Despite its side effects (Roe, 1977; Andersson, 1981), metronidazole is the drug of choice against amebiasis (Nagaraja and Ankri, 2019). However, metronidazole-resistant *E. histolytica* clinical isolates (Bansal et al., 2004; Dario, 2008; Iyer et al., 2017; Victoria-Hernandez et al., 2020) and strains (Abid et al., 2008) have been reported recently, challenging the efforts for amebiasis eradication (Shirley et al., 2019). For this reason, alternative amebiasis treatments are being pursued (Valdes et al., 2014; Ehrenkaufer et al., 2018; Kumar et al., 2018; Mendoza-Figueroa et al., 2018; Mori et al., 2018; Torres-Cifuentes et al., 2018; Valdes et al., 2018; Probst et al., 2019); for example, drugs can be targeted to the species-specific domains present in some *E. histolytica* splicing factors or regulatory noncoding RNAs.

**Figure 1:**
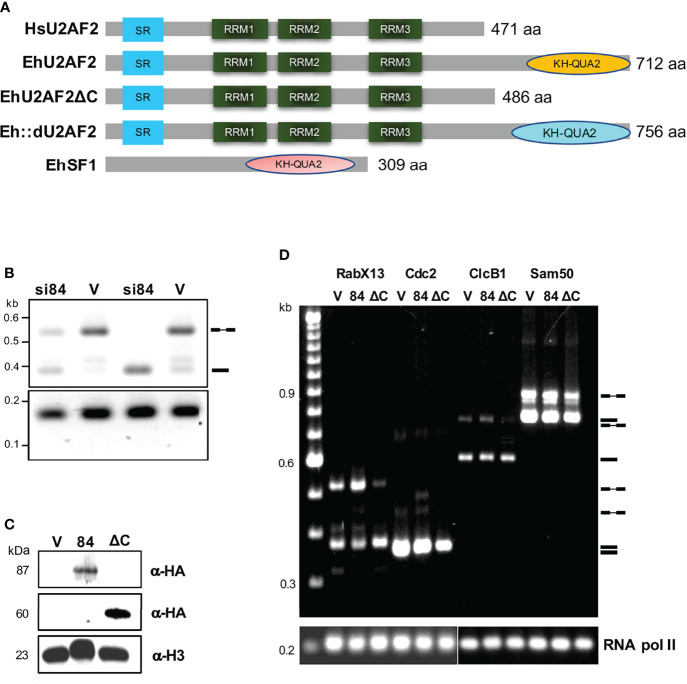
*In vivo* splicing assays of amoeba transformants. **(A)** Diagram of the protein domains of the human U2AF2, EhU2AF2, EhU2AF2DC, Eh::dU2AF2, and EhSF1 splicing factors. The serine-arginine rich, the three RRM, and the KH-QUA2 domains are indicated. **(B)** The RabX13 transcripts profile was monitored by RT- PCR, from silenced (si84) and control psAP-Gunma (V) transfectants selected with 1 and 5 μg/ml of G418 (unspliced/IR variants, 510 bp; spliced form, 374 bp). **(C)** The expression of recombinant proteins in the transfectants was monitored by western blots using anti-HA antibodies. An anti-histone antibody was used as a loading control. Molecular weights are indicated in kDa. **(D)** RT-PCR assays for RabX13, Cdc2, ClcB1, and Sam50 transcripts were performed for the empty pEhExHA vector (V), EhU2AF2 (84), and EhU2AF2DC (DC). On the right are the symbols for mRNA and pre-mRNA, and the migration of molecular weight markers is shown to the left; the RNA pol II transcript was used as a loading control.

The presence of introns, and spliceosomal components in deep**-**branching eukaryotes, including *Entamoeba histolytica*, is well documented, particularly on the DExH-box RNA helicases that proofread the splicing process (Miranda et al., 1996; Hernandez-Rivas et al., 2000; Nixon et al., 2002; Vanacova et al., 2005; Chen et al., 2008; Davila Lopez et al., 2008; Lopez-Camarillo et al., 2008; Marchat et al., 2008; Valdes et al., 2014; Torres-Cifuentes et al., 2018; Valdes et al., 2018). Previously, we identified 36 *E. histolytica* splicing factors in pre-mRNP complexes assembled *in vivo* (Valdes et al., 2014). Among these factors, the Entamoeba EhU2AF1 (EHI-192500) and EhU2AF2 (EHI_098300) orthologs were identified, of 33 and 84 kDa, respectively. Unlike the human and yeast orthologs, the carboxyl terminus domain of EhU2AF2 has extra 226 amino acids comprising two motifs, a heterologous ribonucleoprotein domain with homology to the hnRNP K (KH) motif, followed by an alpha helix QUA2 motif, which are critical for RNA-binding interactions (**Figure 1A**). SF1 also has KH-QUA2 motifs, and both join to specifically interact with the residues of the BS, contributing to the recognition and forming an extended interface with RNA (Teplova et al., 2013). The KH-QUA2 (KQ, for short) domain is a principal feature of STAR proteins (signal transduction and activation of RNA), which bind to RNA or ssDNA, and is importantly involved in transcriptional and translational regulation, along with other cellular processes (Valverde et al., 2008), mainly *via* KH motif-mediated protein dimerization (Feracci et al., 2016). The KQ domain of EhU2AF2 (84KQ) is not fully conserved in other Entamoeba species. In the non-pathogenic human commensal *E. dispar*, it is 44 aa larger, and it is absent or truncated in the non-pathogenic human parasite *E. moshkovskii*, in the parasite of non-human primates *E. nuttalli*, and in the reptilian parasite *E. invadens*, respectively.

*E. histolytica* transcriptomes have shown the association of genes involved in RNA metabolism with the amoebic virulence (Meyer et al., 2016; Weber et al., 2016). Among the upregulated genes in amoebic liver abscesses are the splicing factors EhU2AF2 and a paralog of EhSF3B1 (Valdes et al., 2014). For this reason and because of its unusual protein structure, here we investigated how EhU2AF2 functions in pre-mRNA processing impact the virulence of the parasite. We found that 84KQ is involved in splicing inhibition/intron retention of several virulence and non-virulence-related genes. The 84KQ domain interacts with the same domain of the constitutive splicing factor SF1 (SF1KQ) preventing splicing complex E to A transition. Surprisingly, the deletion of the 84KQ domain in U2AF amoeba transformants increased splicing and enhanced the *in vitro* and *in vivo* virulence phenotypes. We conclude that the interaction of the 84KQ and SF1KQ domains, probably involving additional factors, tunes down Entamoeba virulence by favoring intron retention.

## Material and Methods

### Entamoeba Cultures

Axenic cultures of *E. histolytica* trophozoites (strains HM-1: IMSS and G3) were incubated at 37°C in 13 × 100-mm screw-capped Pyrex glass tubes or plastic culture flasks in TYI-S-33 medium as described (Diamond et al., 1972; Diamond et al., 1978; Diamond et al., 1995). For growth curves, 10^4^ trophozoites were seeded in triplicate cultures and cells were counted at 6, 12, 24, 48, 72, and 96 h of incubation. Experiments were done for three biological replicates. Doubling time was calculated from exponentially adjusted log growth curves.

### Plasmid Constructs and Amoeba Transformants

EhU2AF2 (EHI_098300) was amplified by RT-PCR using primers flanked with the SmaI and XhoI restriction sites, just before the AUG and after the stop codons, respectively (for detailed PCR conditions and primer sequences, see **Supplementary Table 1**). To obtain plasmid pHA-U2AF2, the amplified 2,159-bp fragment was cloned into the SmaI/XhoI-digested pEhExHA expression vector. To obtain the pHA-U2AF2ΔC plasmid, a 1,468-bp fragment of the N-terminus of EhU2AF2 was amplified by PCR using the pHA-U2AF2 plasmid as a template. Because the ends of the sense and antisense oligos included SmaI and NheI restriction sites, respectively, the fragment was compatible with the SmaI/XhoI-digested pEhExHA vector. To obtain the *E. histolytica*::*E. dispar* U2AF2 fusion protein (Eh::dU2AF2), the KH-QUA2 domain was amplified by RT-PCR using total RNA from *E. dispar* (EDI_161905), and sense and antisense oligos carrying NheI and XhoI restriction sites, respectively. The SmaI/NheI-digested EhU2AF2ΔC fragment was ligated to the NheI/XhoI-digested EdKH-QUA2 fragment in a 1:3 ratio with T4 ligase (Invitrogen No. catalog 15224-017) at 16°C according to the manufacturer’s instructions. The ligation product (Eh::dU2AF2) was amplified using appropriate oligos, digested with SmaI and XhoI enzymes, purified, and ligated to the SmaI/XhoI-digested pEhExHA plasmid (**Supplementary Figure 1**). To obtain the His-84KQ and GST-SF1KQ recombinant proteins, the KH-QUA2 domains of EhU2AF2 and EhSF1 (EHI_193510) were amplified by PCR from genomic DNA. Respectively, fragments of 384 and 360 bp were amplified. The 5′ end of the forward primers contained BamHI sites, and the reverse primers contained XhoI sites immediately after the stop codons. The fragments were cloned into the same sites in plasmids Pet28a and pGex 6p1, respectively, to obtain the pHis-84KQ and pGST-SF1KQ plasmids. For EhU2AF2 silencing, the first 400 nt of the gene was amplified with primers flanked by StuI and SacI restriction sites and cloned into the psAP2-Gunma vector giving rise to plasmid psi84 which was transfected in the G3 strain. All constructs were verified by sequencing. The transformation and establishment of amoeba transformants were carried out as described (Valdes et al., 2014).

### RT-qPCR and Intron Retention Index Calculation

KAPA SYBR Fast Universal One-Step RT-qPCR Kit (Sigma-Aldrich, St. Louis, MO, USA) was used for quantitative RT-PCR with 10 ng of cDNA input in 10 µl. EhRNA Pol II (EHI_056690) was used as a normalizer to calculate all transfectants’ relative expression based on the Livak method (Livak and Schmittgen, 2001). PCR conditions and primer sequences are shown in **Supplementary Table 1**. Intron retention index calculation was carried out as previously described (de la Mata et al., 2010) [100 × intron retention/intron retention + mRNA]. Values represent the additional % with respect to 100 in empty vector transformants.

### Migration Test

The transfected trophozoites were fasted in serum-free TYI-S-33 medium for 3 h and subsequently centrifuged at 1,000 RPM for 5 min and washed once with serum-free TYI-S-33 medium. Subsequently, they were counted in a Neubauer chamber and 7.5 × 10^4^ trophozoites were placed in the upper part of a transwell (5-μm-diameter pore, Costar) in incomplete serum-free medium; 500 μl of BSA was placed in the lower part of the transwell and incubated for 3 h at 37°C. Finally, the upper part of the plate was removed and the trophozoites that migrated to the lower part of the transwell were collected, and the corresponding quantification was carried out (Rastew et al., 2015). Results are expressed as the percent of total cells that migrated. The experiment was carried out in triplicate and three independent experiments.

### Liver Abscesses

The study of hepatic amebiasis in hamsters was carried out in accordance with the International Standards for the Care and Use of Laboratory Animals (NOM 062-Z00-1999). Three groups of five male hamsters (*Mesocricetus auratus*), 2 months old, with an average weight of 80 ± 10 g, were fasted 24 h before inoculation of the trophozoites. The hamsters were intraperitoneally anesthetized with sodium pentobarbital (Anestesal, Smith Kline, Mexico City, Mexico), and then they were inoculated intrahepatically with 1 × 10^6^ trophozoites in 200 μl of incomplete TYI-S-33 medium, from a 48-h culture. The hamsters were humanely euthanized 7 days after infection, and the abscess percentage of each liver was determined manually by weighing the entire liver and separately the area with and without abscesses to approximate tissue damage. Three independent *in vivo* virulence experiments were carried out. Animal handling was carried out according to the Mexican Official Norm NOM-062-ZOO-1999 for the production, care, and use of laboratory animals (UPEAL Ethics Committee Approval Protocol No. 0184-16; Section Ethics approval and consent to participate).

### Erythrophagocytosis

RBCs (red blood cells) ingested by amoeba were quantified by a colorimetric method essentially as described (Sahoo et al., 2003). 10^7^ RBCs, washed in PBS and TYI-33, were incubated with 5^4^ trophozoites for 15 and 30 min at 37°C in 1 ml of culture medium. Then, amoebae and RBC were pelleted and non-engulfed RBCs were lysed with chilled distilled water. The cell suspension was pelleted twice for 2 min at 1,000 × *g* and resuspended in formic acid to lyse the amoebae containing engulfed RBC. Samples were measured spectrophotometrically at 397 nm against a formic acid blank.

### Statistical Analyses

The values represent the average of at least three independent experiments, significance being (^ns^,*,**,***,^&^) non-significant, *P* < 0.01, 0.001, 0.0001, 0.0005. The RT-qPCR data were analyzed using the 2^-ΔΔCT^ method. GraphPad Prism v.5 for Windows (GraphPad Software, San Diego, CA, USA) was used to analyze results, using Student’s t-test and Kruskal–Wallis test.

### Purification of Recombinant Proteins

*E. coli* BL21 (DE3) was transformed with plasmids pHis-84KQ and pGST-SF1KQ. A single colony each was grown in 0.5 l of TB broth with 25 µg/ml of kanamycin or 100 µg/ml ampicillin, respectively. When the cultures reached an O.D. of 0.7 at 600 nm, IPTG was added to 0.5 mM and cultures were shaken at 37°C for 4 h. Bacteria were harvested by centrifugation and were suspended either in 5 ml of imidazole lysis buffer (50 mM Tris–HCl, pH 7.5, 500 mM KCl, 15 mM imidazole) or in binding buffer (PBS: 140 mM NaCl, 2.7 mM KCl, 10 mM Na_2_HPO_4_, 1.8 mM KH_2_PO_4_, pH 7.3). In both cases, one pill of Complete Mini was added. Cells were lysed with 6× 30-s rounds of sonication at 75% amplitude on ice. Insoluble material was removed by centrifugation at 12,000 rpm for 20 min. His-84KQ supernatant was mixed with 1 ml of Ni-NTA QIAGEN beads, and the resin was recovered in a column and rinsed with 10 ml of wash buffer (50 mM Tris–HCl, pH 7.5, 500 mM KCl, 20 mM imidazole). Five 1-ml elution (wash buffer with 300 mM imidazole) fractions were collected and monitored by SDS-PAGE. Positive fractions were dialyzed overnight in 2 ml of dialysis buffer (20 mM Tris–HCl, pH 7.5, 100 mM KCl, 10% glycerol). GST-SF1KQ supernatant was collected in GSTrap FF columns and washed 5× with binding buffer. Proteins were eluted with 50 mM Tris–HCl, 10 mM reduced glutathione, pH 8.0, and analyzed as before. Proteins were quantified by Qubit and stored at -70°C.

### Far Western Blot

Total protein extracts (20 µg) of amoeba transformants were fractionated in 12% SDS-PAGE gels and electroblotted onto nitrocellulose and stained with 1% Ponceau Red for 5 min. The membranes were blocked with PBS–0.02% Tween 20–5% skimmed milk for 1 h at room temperature and were incubated with 10 mM of the His-84KQ or the GST-SF1KQ recombinant proteins as before. Membranes were washed 3× with PBS–0.02% Tween 20 and were developed with the appropriate antibody as described (Ortuno-Pineda et al., 2012).

### EMSA, UV-CL, Formaldehyde-CL, and Crosslink Pulldown (CLPD)

*E. histolytica* or yeast BS probes were end-labeled with [γ-^32^P]-ATP and T4 Polynucleotide Kinase and used in electrophoretic mobility shift and UV-crosslinking assays essentially as described (Ortuno-Pineda et al., 2012). Protein mixes were treated with 2% formaldehyde for 15 min, and reactions were stopped with glycine 250 mM in PBS. Crosslinked reactions were heat-denatured in 2× SDS-PAGE loading buffer. For CLPD assays, after UV- or formaldehyde-CL, 20 µg of GST beads was added and the volume was brought to 60 µl with binding buffer. The reaction was incubated at 4°C for 2 h, and beads were centrifuged at 1,000 rpm for 5 min. After 3× PBS washes, samples were resuspended in 2× loading buffer and analyzed by SDS-PAGE and blotting. Membranes were autoradiographed and rehydrated for Western blot analysis.

## Results

### The Role of the EhUA2AF2 KH-QUA2 Domain on Splicing and the Parasite’s Virulence

To explore the role of EhU2AF2 in splicing of amoeba pre-mRNAs, *in vivo* splicing assays were performed with amoeba transformants where EhU2AF2 was silenced or overexpressed, monitoring the RabX13 pre-mRNA splicing pattern (**Figure 1**). The control psAP2Gunma transformants (V) produced substantial amounts of unspliced/intron retained (IR) and fewer spliced variants. Surprisingly, the silenced pGsi84 transformants (si84) increased RabX13 mRNA, indicating that EhU2AF2 is involved in splicing inhibition, thus increasing IR (**Figure 1B**). To identify the domain involved in IR, we analyzed amoeba transformants overexpressing the 84KQ-deleted EhU2AF2 (ΔC) compared to the full-length EhU2AF2 (84). The expression of each recombinant protein was verified by Western blot in protein extracts of the amoeba transformants (**Figure 1C**). Total RNA was extracted, and RabX13, Cdc2, ClcB1, and Sam50 transcript variants were monitored. In agreement with our previous finding, mRNAs accumulated in ΔC transformants, accompanied by reduction of unspliced/IR variants, indicating that the 84KQ domain is required for the EhU2AF2 splicing inhibition function (**Figure 1D**).

To gain insights into the effect of EhU2AF on splicing, we analyzed the IR index (IR abundance divided by mRNA+IR) obtained from RT-qPCR of *in vivo* splicing assays of several virulence-related genes. Compared to 100% in the empty vector transfectants, the overexpression of EhU2AF2 increased the IR of the analyzed transcripts as an inverse function of intron size (**Table 1**; introns were ordered according to their size), and in U2AF2ΔC amoebas most transcripts showed an IR mean reduction of 18.7 points (ranging from 0 to 32). These data were compared to amoeba transformants expressing the chimeric RNA-binding domains of EhU2AF2 followed by the KH-QUA2 domain of the non-virulent human commensal *E. dispar* (Eh::d) (**Figure 1A**). Since the EdKQ domain is larger, we predicted that such domain would not affect splicing to the same extent as 84KQ, indicating a salient role of 84KQ in the virulent phenotype of the parasite. As expected, the mean reduction of the IR indexes obtained with the Eh::d chimera was less pronounced (11.3 points, ranging from -2 to 28), supporting the inhibitory role of the 84KQ domain on splicing. Interestingly, different results were obtained for the transcripts of locus EHI_014170. The intron of these transcripts has a very short Py tract in front of the 3′ss. It is possible that these transcripts may be poorly recognized by EhU2AF2 making the 84KQ domain function ineffective. These data open the possibility for additional factors to participate together with 84KQ and SF1KQ in the partial splicing inhibition process. So far, our data indicate that the 84KQ domain of EhU2AF2 diminishes splicing efficiency.

**Table 1:** IR index*^a^* of different amoeba transformants.

Gene ID	84	ΔC	Eh::d	Intron length (nt)	PyAG*^b^*
EHI_169670 i2	53	35	40	327	70
EHI_192510	40	28	42	256	60
EHI_014170	34	34	28	251	30
EHI_169670 i1	59	27	31	152	60
EHI_083590	53	36	43	73	60
EHI_098590	72	47	55	69	70
EHI_042870	67	40	60	50	70

^a^IR/IR+splicing, mean values of triplicates (% over 100 in empty vector transformants).

^b^Percent of pyrimidines within the 15 residues upstream of the 3′ss dinucleotide.

EhU2AF2 is overexpressed in amoebae recovered from liver abscesses (Weber et al., 2016), suggesting that this splicing factor plays a major role in virulence. To link the function of EhU2AF2 with virulence-related traits, we measured the proliferation curves, erythrophagocytosis, transwell migration, and liver abscess formation in hamsters in the aforementioned amoeba transformants. Interestingly, with respect to the empty vector transformants, a sharp increase in all traits analyzed was observed in the 84 amoeba transformants (**Table 2**). Amebic liver abscess formation showed the highest increase, suggesting enhanced virulence of the 84 strains. As expected, compared to the 84 transformants, the growth rate of ΔC transformants was reduced by half, and that of the Eh::d chimera was reduced by 33% (**Table 2**) suggesting low and moderate virulence of these strains, respectively. Complementary results were observed for the remaining virulence factors (**Table 2**); particularly, compared to empty vector transformants (V), in ΔC erythrophagocytosis increased by 40%–70%, the amoebas migrated four times more, and liver abscess formation increased 10-fold (**Table 2** and **Supplementary Figure 2**). These results suggest that the KH-QUA2 domain, perhaps with the aid of additional factors, is involved in the downregulation of the parasite’s virulence.

**Table 2:** Virulence factors of amoeba transformants.

Factor		V	84	ΔC	Eh::d
Growth rate (doubling time, h)*^a^*		12.1	12.6^ns^	6.7^***^	8.4^***^
Erythrophagocytosis (OD_397nm_)*^b^*	15 min	0.7	1.0^ns^	1.2^**^	1.2^**^
	30 min	1.3	1.4^ns^	1.8^*^	1.4^ns^
Transwell migration (% of total cells)*^c^*		4	10^***^	15^***^	12^***^
Liver abscesses (% of weigh)*^d^*		4	18^**,&^	40^***,&^	28^***,&^

^a^Calculated from exponentially adjusted log growth curves.

^b^Colorimetric estimations of engulfed RBC.

^c^Upper chamber inputs 7.5 × 10^4^ trophozoites.

^d^Inoculum 10^6^ trophozoites.

Statistically significant (Student’s t-test *P < 0.01; **P < 0.001; ***P < 0.0001; ns, nonsignificant; and Kruskal–Wallis, ^&^P < 0.0005) compared to empty vector transformants.

### EhUA2AF2 and EhSF1 KH-QUA2 Domain Interaction

**Figure 2:**
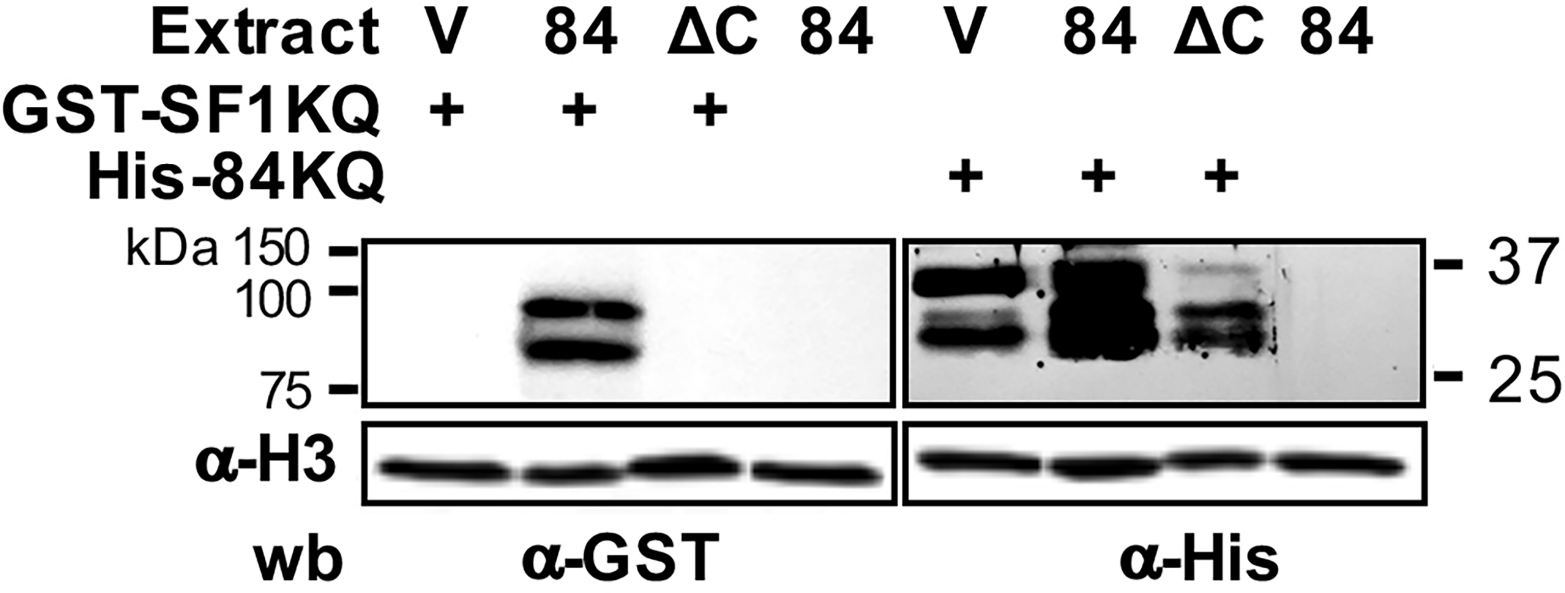
Mutual binding of the KH-QUA2 motifs of EhU2AF2 and EhSF1. Protein extracts from empty vector control (V), EhU2AF2 (84), and EhU2AF2ΔC (ΔC) amoeba transformants were electroblotted onto nitrocellulose and incubated without (-) or with the GST-SF1KQ or the His-84KQ recombinant proteins. Blots were then incubated with anti-GST or anti-His antibodies and developed with chemiluminescence. Anti-histone 3 antibodies were used as loading controls. Migration of molecular weight markers is shown to the left.

Next, we studied the molecular mechanisms by which the 84KQ domain impacts splicing and virulence. Since the 84KQ domain is very similar to SF1KQ, responsible for BS recognition, we hypothesized that the splicing inhibition exerted by EhU2AF could be the result of the competition of these domains for the BS, or by steric hindrance due to KH–KH domain dimerization, as observed in Quaking protein (Feracci et al., 2016). In any case, we would expect blocking splicing complex E to A transition. To discern between these possibilities, we produced recombinant GST-tagged SF1KQ (GST-SF1KQ) and His-tagged 84KQ (H6-84KQ) (**Supplementary Figure 3**) proteins that were used in far Western, EMSA, UV and formaldehyde crosslinking, and CLPD (crosslinking pulldown) experiments. To test that both domains interact with each other, total protein extracts from V, 84, and ΔC amoeba transformants were incubated with the recombinant GST-SF1KQ or H6-84KQ proteins. Then, blots were probed with anti-GST or anti-His antibodies, respectively, and developed with chemiluminescence. The SF1KQ domain recognized an 84-kDa protein doublet in the 84 transformants and to a lesser extent the top band in the V transformants. As expected, no signal was detected in the ΔC amoebas (**Figure 2**, lane 2). Correspondingly, a 35-kDa protein was strongly recognized by the H6-84 protein in the V and 84 amoeba transformants. The signal was greatly reduced in the ΔC transformants (**Figure 2**, lane 7). No signals were observed when no recombinant proteins were added to the 84 transformants. This was corroborated by the anti-Histone antibody loading controls. These results indicate that both KQ domains interact with each other.

**Figure 3:**
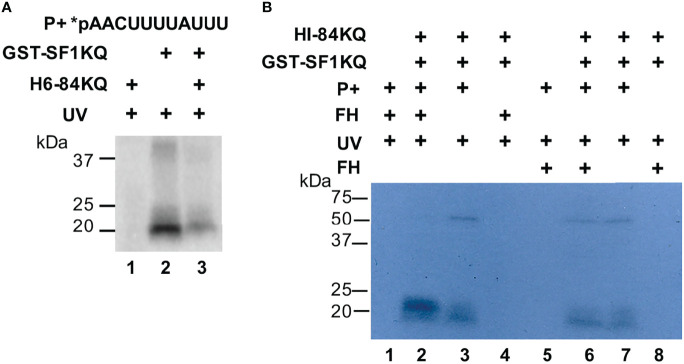
RNA-independent interaction of the EhU2AF2 and EhSF1 KH-QUA2 motifs bound to the BS. **(A)** GST-SF1KQ protein was incubated without or with the H6-84KQ protein before UV-crosslinking to radiolabeled *E*. *histolytica* BS probes (5′-AACUUUUAUUU-3′). An H6-84KQ plus BS control was performed. **(B)** UV- and formaldehyde-crosslinking assays were carried out in the order specified on top of the autoradiogram. The migration of molecular weight markers is shown to the left.

We used the intronic radiolabeled BS sequence (*pAACUUUUAUUU) of the RabX13 gene from *E. histolytica* in EMSA and UV-crosslinking experiments to verify that it is recognized by the SF1KQ domain and to explore the interactions between the 84KQ and SF1KQ motifs. Increasing amounts of either 84KQ or SF1KQ with constant quantities of the corresponding protein-supershifted BS-SF1KQ complexes (**Supplementary Figure 4**) indicate a strong interaction between both proteins in the BS complex. Since the Prot-param-estimated pI of 84KQ is 4.02, we did not expect this domain to bind to the BS at physiological pH; however, it did crosslink to the BS at pH 4 (**Supplementary Figure 5**), demonstrating that in our experiments, 84KQ binds to the SF1KQ already bound to the BS. In agreement with this, soluble 84KQ inhibited the interaction of the SF1KQ domain with the BS when added before protein–RNA complexes are irradiated (**Figure 3A**, compare lanes 2 and 3). So far, evidence indicates that both KQ domains interact in solution. To test whether the 84KQ domain recognizes the SF1KQ bound to the BS, we used formaldehyde crosslinking (protein–protein) before or after UV crosslinking (protein–RNA). Compared to no formaldehyde controls (lanes 3 and 7), the signal of the SF1KQ crosslinked to the BS disappeared when formaldehyde was added before UV irradiation (**Figure 3B**, lane 2); however, the signal remained when formaldehyde was added after irradiation (**Figure 3B**, lane 6), indicating that the 84KQ domain also interacts with prebound SF1 to the BS. Altogether, these results indicate that the 84KQ and SF1KQ domains mutually interact in solution and when bound to the BS RNA.

### Mechanistic Outcome of the EhUA2AF2-EhSF1 KH-QUA2 Domain Interaction

**Figure 4:**
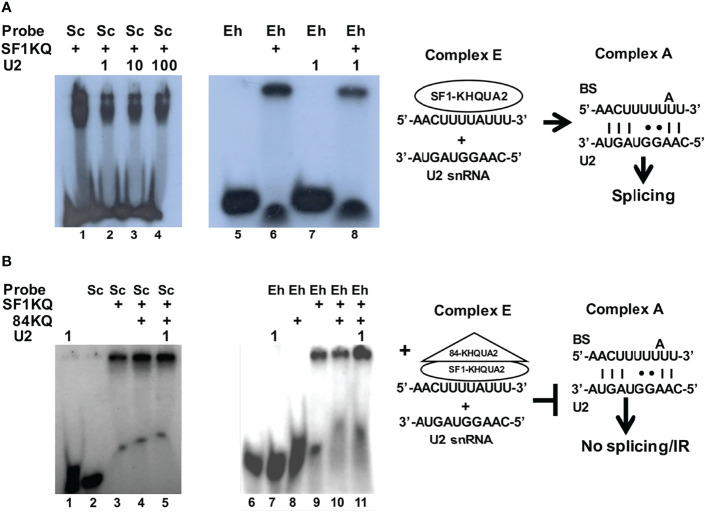
The KH-QUA2 motifs of EhU2AF2 inhibit splicing complex E to A transition. **(A)** EMSAs were carried out with 50 µg of recombinant EhSF1-KQ and 200 fmol of either the yeast control BS (5′-UAUACUAACAA-3′) or the amoeba-specific EhRabX13 intron BS. After 20 min of incubation, *E. histolytica* cold U2 snRNA (1×, 10×, or 100× molar excess; 5′-CAAGGUAGUA-3′) was added to the reactions and was further incubated for 10 min. **(B)** EMSAs were carried out as described, except that after 20 min of incubation, 50 µg of the recombinant 84KQ protein was added to the reactions before the addition of 1× molar excess of cold U2 snRNA. To the right of each assay appears a schematic interpretation of the results leading to productive splicing **(A)** or splicing inhibition/intron retention **(B)**, respectively.

The spliceosome is recruited on the pre-mRNA substrate as it is transcribed; the transition from splicing complex E to A involves first the KH-QUA2 domain-mediated stabilization of SF1 and U2AF2 interaction on the BS (Selenko et al., 2003; Zhang et al., 2013) followed by the displacement of SF1 as the U2 snRNA hybridizes with the BS (Zhang et al., 2019). We hypothesized that the 84KQ domain could overstabilize the SF1KQ–BS interaction obstructing SF1 for U2 snRNA substitution. EMSA assays were designed to test this. Radioactive probes harboring the yeast canonical BS and the EhRabX13 intron BS were shifted with the recombinant SF1KQ domain (**Figures 4A, B** **)**. The shifted signals were reduced by half in the presence of the U2 snRNA (lanes 2–4 and 8), indicating that the latter partly displaced SF1 from the BS. Notably, such displacement was inhibited by the 84KQ domain (**Figure 4B**, lanes 5 and 11). As expected, the probe-, U2 snRNA-, and 84KQ-only controls showed no mobility shift. These results suggest that the 84KQ domain overstabilizes SF1 interaction with the BS, thus blocking splicing complex E to A transition and increasing the IR rate.

## Discussion

Here we describe a novel function of the atypical splicing factor U2AF2 in *E. histolytica*. Different from other orthologs, the carboxy terminus of EhU2AF2 harbors a KH-QUA2 domain in its 226-amino acid-long extension. This domain appears to grip the interaction of SF1 with the branch point (probably with the aid of additional factors), thus inhibiting splicing and downregulating the virulence of the parasite.

It is well known that in model mammalian and yeast genes, U2AF2 defines functional 3′ss, yet it was until recently that this was described to occur also *in vivo* (Shao et al., 2014). Also, U2AF2 is involved in alternative splicing modifying the selection of different 5′ss (Forch et al., 2003; Ortuno-Pineda et al., 2012), and when U2AF2 binds additional sites within the intron, it helps select alternative 3′ss (Shao et al., 2014). In our silencing and overexpression experiments, we expected to observe the involvement of EhU2AF2 in splicing (**Figure 1**), but the observation of higher IR in shorter introns was unexpected (**Table 1**). Two possible explanations might account for these results: first, the AG-dependent weak introns with very short Py (ca. 4 U residues) require the U2AF1/2 dimer to activate the 3′ss and splicing; in strong AG-independent introns with long Py (≤ 8 U residues), U2AF2 suffices for activation (Wu et al., 1999). Concurrently, closed U2AF2 conformation recognizes weak Py and the more stable open conformation binds to strong Py (Corsini et al., 2007; Mackereth et al., 2011). In addition, this event might be aided by EhTIA which is able to bind strong Py, considering that EhTIA was immunoprecipitated along with EhU2AF2 in spliceosomal complexes (Valdes et al., 2014). Second, to initiate spliceosome assembly during E to A complex transition on short introns with short Py, the U2AF-homology motif (UHM) of SPF45 competes with that of U2AF2 for binding to the UHM-ligand motif (ULM) of the U2 snRNP protein SF3B1. Apparently, splicing of human short introns depends on SPF45 but not the U2AF1/2 dimer (Fukumura et al., 2021). So far, we have no other examples of introns with short Py, thus weakening our arguments. However, we are conducting RNA-seq experiments to identify the collection of transcripts where EhU2AF2 binds and to explore how the absence of EhU2AF2 impacts overall splicing efficiency and gene transcription regulation. This last point is relevant since our results suggest increased gene expression in some of the tested transcripts in U2AF2ΔC amoebas (**Supplementary Figure 6**).

We observed that deletion of the KH-QUA2 domain increased the splicing efficiency of virulence-related transcripts indicating a splicing inhibitory function of such domain (**Figure 1** and **Table 1**). This function was unforeseen; however, recently it has been described that QK1 (Quaking 1) inhibits the splicing cassette exons (Liao et al., 2021). QK1 is a member of the STAR domain-containing proteins, a domain composed of QUA1, KH, and QUA2 motifs that enable them to bind RNA recognition elements *in vivo* (Teplova et al., 2013) and to dimerize through their KH motif (Feracci et al., 2016). Therefore, QK1 may inhibit splicing either by RNA binding or by interaction with other spliceosomal proteins. Furthermore, QK1 may inhibit forward splicing of certain exons inducing back-splicing and producing circular RNAs (Conn et al., 2015). We are conducting experiments to identify the amino acids of the EhU2AF KH motif required to interact with the EhSF1 KH motif (**Supplementary Figure 7**).

Our most insightful finding, with profound biological consequences, is the role of 84KQ on the attenuation of virulence due to splicing inhibition (**Table 2**). While in humans, U2AF2 has been associated with alternative splicing in transformed cells (Shao et al., 2014), the ortholog EhU2AF2 has been associated with virulence because it is overexpressed in amoebas recovered from liver abscesses (Weber et al., 2016). A similar association has been drawn for the U2 snRNP SF3B1 ortholog EHI_085470 (Meyer et al., 2016) and its paralog EHI_049170 (Valdes et al., 2014) which are present in virulent *E. histolytica* strains. Our work revealed an important role of EhU2AF2 beyond the sole overexpression of this virulent-specific protein. Few reports describe splicing factors’ activity modifying parasite virulence. In contrast to our findings, in the rice blast fungus *Magnaporthe oryzae*, deletion of either one of two splicing factors—the glycine-rich protein MoGrp1 and the Prp19 associated factor Cwf15—is sufficient to impair fungal development and virulence, suggesting that they upregulate both intron splicing and fungal virulence (Gao et al., 2019; Liu et al., 2021). Splicing inhibition-induced virulence attenuation has been observed in a natural isolate of the chestnut blight *Cryphonectria parasitica*. This strain carries an insertion of a maturase-defective A1 group II intron named InC9 (a 971-bp DNA element) in the first exon of the small subunit rRNA gene. Because InC9 is inefficiently spliced, very low amounts of mitochondrial ribosomes are produced, lowering mitochondrial protein synthesis and lacking components of the cytochrome-mediated respiratory pathway, thus attenuating the fungal virulence (Baidyaroy et al., 2011). In *E. histolytica*, the 84QK-driven attenuation of virulence due to splicing inhibition affects most virulence-related phenotypes (growth rate, erythrophagocytosis, chemotaxis, and liver abscess formation), suggesting that EhU2AF2 modifies the splicing efficiency of a wide set of genes; we are currently exploring this issue. This is in agreement with previous findings showing the overexpression of EhU2AF2 in trophozoites recovered from hamsters’ liver abscess (Weber et al., 2016), since these amoebas display more virulent traits than wild-type strains but are less aggressive than the amoebas without the 84KQ motif. It is widely accepted that increased parasite growth leads to greater virulence (e.g., harm they cause to their host) (Anderson and May, 1982; Frank, 1996). This model has been recently challenged because opportunistic parasites and encysting intracellular parasites behave differently (Frankel et al., 2007; Brown et al., 2012; Leggett et al., 2017). Here we found that EhU2AF modulates splicing and attenuates the virulence of wild-type *E. histolytica* in agreement with non-opportunistic non-intracellular organisms; in other words, splicing modulation could lead to asymptomatic colonization and limit invasion.

We also explored the mechanism by which 84KQ blocks the splicing complex E to A transition. Several experiments suggested that the 84KQ and SF1KQ domains might dimerize. In solution, they interacted with each other (**Figures 2** and **3**), 84KQ prevented SF1KQ–BS interaction, and 84KQ could bind to SF1KQ when complexed with BS RNA probes (**Figure 3**). These data support the notion that the KH-QUA2 domains of EhU2AF2 and SF1 might form dimers either through KH–KH interactions as observed in other STAR proteins (Feracci et al., 2016) or *via* UHM–ULM interactions (Berglund et al., 1998; Rain et al., 1998; Rudner et al., 1998a; Rudner et al., 1998b). More importantly, the 84KQ domain impedes the substitution SF1 for U2 snRNA at the BS (**Figure 4**), suggesting a significant role of the 84KQ domain in complex E to A transition blockage. The incomplete signal reduction by the U2 snRNA may be due to the lack of splicing components, Prp5 and UAP56 RNA helicases for example, in the reaction. It has been reported that during E to A transition Prp5 purges U1 excess from the 5′ss and U1 alters mRNA properties (Hodson et al., 2012); therefore, it is likely that UAP56 is the missing helicase in our conditions. The observed partial signal reduction could also be due to the possible activity loss of the recombinant protein extracts, since we required considerable amounts (50 μg) in our experiments.

In yeasts, E complex formation involves proteins that bridge and stabilize the splice sites (Larson and Hoskins, 2017). In higher eukaryotes, the interaction between the E complex splicing factors U2AF1, U2AF2, and SF1 cooperatively defines the 3′ss, where SF1, U2SF2, and U2SF1 bind to the BS, the Py, and the AG dinucleotide of the 3′ss, respectively. They interact with each other through UHM and ULM motifs that enhance the binding affinity to the mRNA (Berglund et al., 1998; Rain et al., 1998; Rudner et al., 1998a; Rudner et al., 1998b). Whereas the UHM of U2AF1 interacts with the ULM in the N terminus of U2AF2 forming a stable U2AF1/2 heterodimer, the UHM (RRM3) of U2AF2 interacts with the ULM at the end of phosphorylated SF1 (Wang et al., 2013). Transition to complex A involves ATP-dependent structural rearrangements of the nascent spliceosome (Lim and Hertel, 2004). Namely, the SF1–BS interaction is replaced by the hybrid formed between the U2 snRNA and the BS, and the SF1–U2AF2 interaction is replaced by the 155-kDa subunit of splicing factor-3b (SF3B1) that now interacts with U2AF2 (Gozani et al., 1998; Das et al., 2000; Corsini et al., 2007; Choi et al., 2020). Furthermore, disruption of the SF3B1–U2AF2 interaction by spliceostatin leads to splicing inhibition (Yoshimoto et al., 2017).

**Figure 5:**
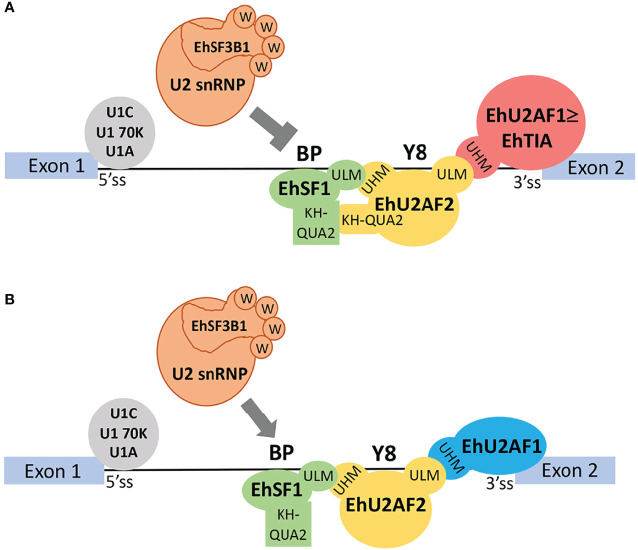
A model of splicing inhibition of EhU2AF2 in amoeba transfectants. **(A)** EhU2AF2 (yellow) is sufficient to activate the 3′ss of AG-independent introns with strong Py tracts (> 6 y). Instead of EhU2AF1, EhTIA1 (red) might help to stabilize the EhU2AF2 interaction with EhSF1 (green). These interactions are mediated by UHM-ULM and ULM-UHM motifs, respectively. EhSF1/BS recognition is gripped by EhU2AF2 *via* their KH-QUA2 domain interactions, thus inhibiting EhSF1 replacement by the U2 snRNP factor EhSF3B1 (orange). **(B)** When EhU2AF2 lacks the KH-QUA2 domain, no additional interactions will occur between EhSF1 and EhU2AF2. Then, EhSF3B1 can replace EhSF1 during splicing complex E to A transition, thus increasing splicing and reducing IR. In the case of AG-dependent introns with weak Py (< 6 y) both EhU2AF2 and EhU2AF1 are required for 3′ss activation. In all cases, 5′ss recognition is carried out by EhU1C, EhU1 70K, and EhU1A.

In *E. histolytica*, all of these factors have been identified in pre-mRNA ribonucleoparticles assembled *in vivo* (Valdes et al., 2014) and all of them have conserved UHM and ULM motifs (**Supplementary Table 2**); therefore, a similar scenario is expected to occur in this parasite. We propose that, in *E. histolytica*, the RRM1 and RRM2 domains of EhU2AF2 recognize the Py tract and that the EhU2AF2 UHM motif interacts with the ULM motif at the N-terminus of SF1 as previously reported (Corsini et al., 2007). Furthermore, 84KQ also interacts with SF1 lengthening the proximity between U2AF2 and SF1. While EhU2AF2-UHM steadies the binding of SF1 to the BS, 84KQ appears to grip such interaction blocking complex E to A transition, thus inhibiting splicing and ultimately negatively impacting the virulence of the parasite. So far, we have only analyzed the EhU2AF2–EhSF1 interaction; however, we cannot discard additional factors, such as the 5′ss activator TIA-1 (Forch et al., 2002), which might also be involved in impeding splicing complex E–A transition. EhTIA-1, like the rest of the previously identified (Valdes et al., 2014) splicing factors involved in complex E–A transition, contains either one or both UHM and ULM motifs (**Supplementary Table 2**); therefore, it is possible that EhTIA-1 competes with EhU2AF2 for SF3B1 binding during complex A formation. In a similar fashion, SPF45 binds to SF3B1 instead of U2AF2 and regulates the alternative splicing of the human *FAS* gene (Corsini et al., 2007). Alternatively, the human splicing factor RBM39 appears to be an EhTIA-1 ortholog and it is known that the binding of RBM39-(UHM) to U2AF2-(ULM) is essential for the selective recognition of Py tracts and U2AF65 entry to the 3′ss. Since the U2AF65–(ULM/UHM)–RBM39 interaction occurs instead of U2AF2–(ULM/UHM)–U2AF1 although with less affinity, it has been proposed that these weak and transitory U2AF2–RBM39 interactions facilitate the more thermodynamically stable U2AF2/1 3′ss recognition. In 3′ss with strong Py, U2AF1 might not be required for 3′ss activation and the U2AF2–RBM39 interactions remain and strengthen the SF3B1–U2AF2–RBM39 complex formation (Stepanyuk et al., 2016; Tari et al., 2019). Because in amoebic introns even the strong Py tracts are short compared to other organisms, we do not expect synergistic EhU2AF2–EhTIA-1 interactions to inhibit splicing; in a similar fashion (but with the opposite outcome), they bind to poly-U tracts and activate 5′ss (Forch et al., 2002; Forch and Valcarcel, 2003; Ortuno-Pineda et al., 2012). Our current understanding of the role of EhU2AF2 in splicing inhibition is that in introns with weak Py EhU2AF2–ULM binds to Eh-U2AF1–UHM, and in introns, with strong Py, it binds to EhTIA-1–UHM. These stable interactions, together with the grip formed between EhU2AF2–UHM and EhSF1–ULM plus both of their respective KH-QUA2 domains, block U2 snRNA entry and the substitution of EhSF1 for EhSF3B1 (**Figure 5A**). When EhU2AF2 lacks the KH-QUA2 domain, this blockage is relieved, irrespective of the Py-tract strength (**Figure 5B**). This issue remains to be confirmed when additional pre-mRNAs with short Py-tracts are identified.

In conclusion, we described a novel function of the atypical EhU2AF2 with a lengthened C-terminus containing a KH-QUA2 domain. Apparently, this domain grips the SF1–BS interaction (probably with the aid of additional factors), thus inhibiting splicing/increasing IR and downregulating the virulence of *Entamoeba histolytica*.

## Data Availability Statement

The raw data supporting the conclusions of this article will be made available by the authors, without undue reservation.

## Ethics Statement

The animal study was reviewed and approved by the Mexican Official Norm NOM-062-ZOO-1999 for the production, care, and use of laboratory animals (UPEAL Ethics Committee Approval Protocol No. 0184-16; Section Ethics approval and consent to participate).

## Author Contributions

GG-B: data curation, formal analysis, investigation, methodology, visualization, writing—original draft preparation; CV: conceptualization, methodology, supervision, writing—review and editing; GG-R: methodology, writing—review and editing; JG-R: methodology; OS-C: methodology, writing—review and editing; EA-L: methodology, writing—review and editing; MAR-R: methodology, writing—review and editing; PT-R: methodology, writing—review and editing; EO: methodology, writing—review and editing; TN: writing—review and editing; JV: conceptualization, funding acquisition, methodology, project administration, resources, supervision, writing—review and editing. All authors contributed to the article and approved the submitted version.

## Funding

This work was funded by CONACYT México (grant CF-2019-194163) and Postgraduate Scholarship to GG-B (730187).

## Conflict of Interest

The authors declare that the research was conducted in the absence of any commercial or financial relationships that could be construed as a potential conflict of interest.

## Publisher’s Note

All claims expressed in this article are solely those of the authors and do not necessarily represent those of their affiliated organizations, or those of the publisher, the editors and the reviewers. Any product that may be evaluated in this article, or claim that may be made by its manufacturer, is not guaranteed or endorsed by the publisher.
