# Supplementary material for: An Unusual U2AF2 Inhibits Splicing and Attenuates the Virulence of the Human Protozoan Parasite Entamoeba histolytica
Source: Front Cell Infect Microbiol. 2022 Jun 17;12:888428. doi: 10.3389/fcimb.2022.888428 (PMC9247205; doi:10.3389/fcimb.2022.888428)
Supplement: Supplementary file 1 [file DataSheet_1.pdf]

Supplementary Material

1 Supplementary Figures and Tables

1.1 Supplementary Figures

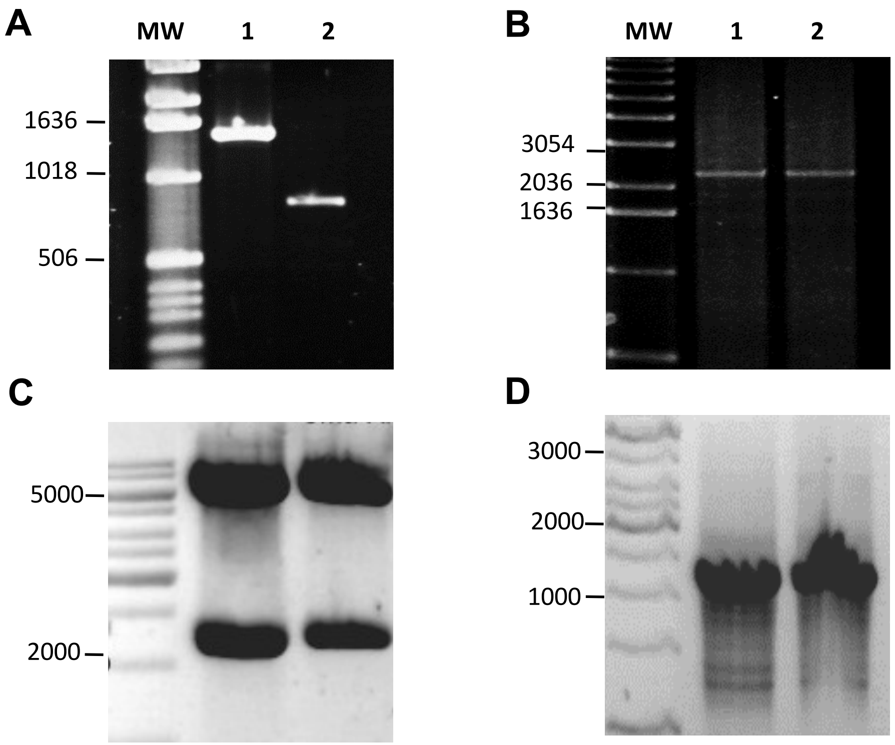

Supplementary Figure 1. Construction of the Eh::dU2AF2 chimera. (**A**) Amplification of the N-terminus domain of EhU2AF2 (lane 1) and the C-terminus domain of EdU2AF2 (lane 2). (**B**) The amplified fragments in A were ligated and amplified by PCR with N- and C-end primers. The ligation products of two independent colonies were tested. (**C**) The pHA-Eh::dU2AF2 plasmid was digested with SmaI and XhoI enzymes liberating the Eh::dU2AF2 insert. (**D**) Reamplification of the Eh::dU2AF2 insert with the end-primers used in (B).

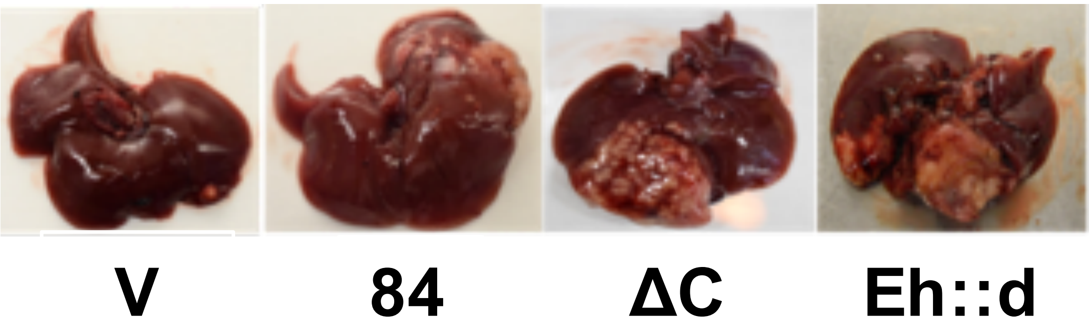

Supplementary Figure 2. Amoebic liver abscesses produced by amoeba transformants. Photographs of representative experiments were taken after the whole-liver weigh assessment and before the isolation of the abscesses.

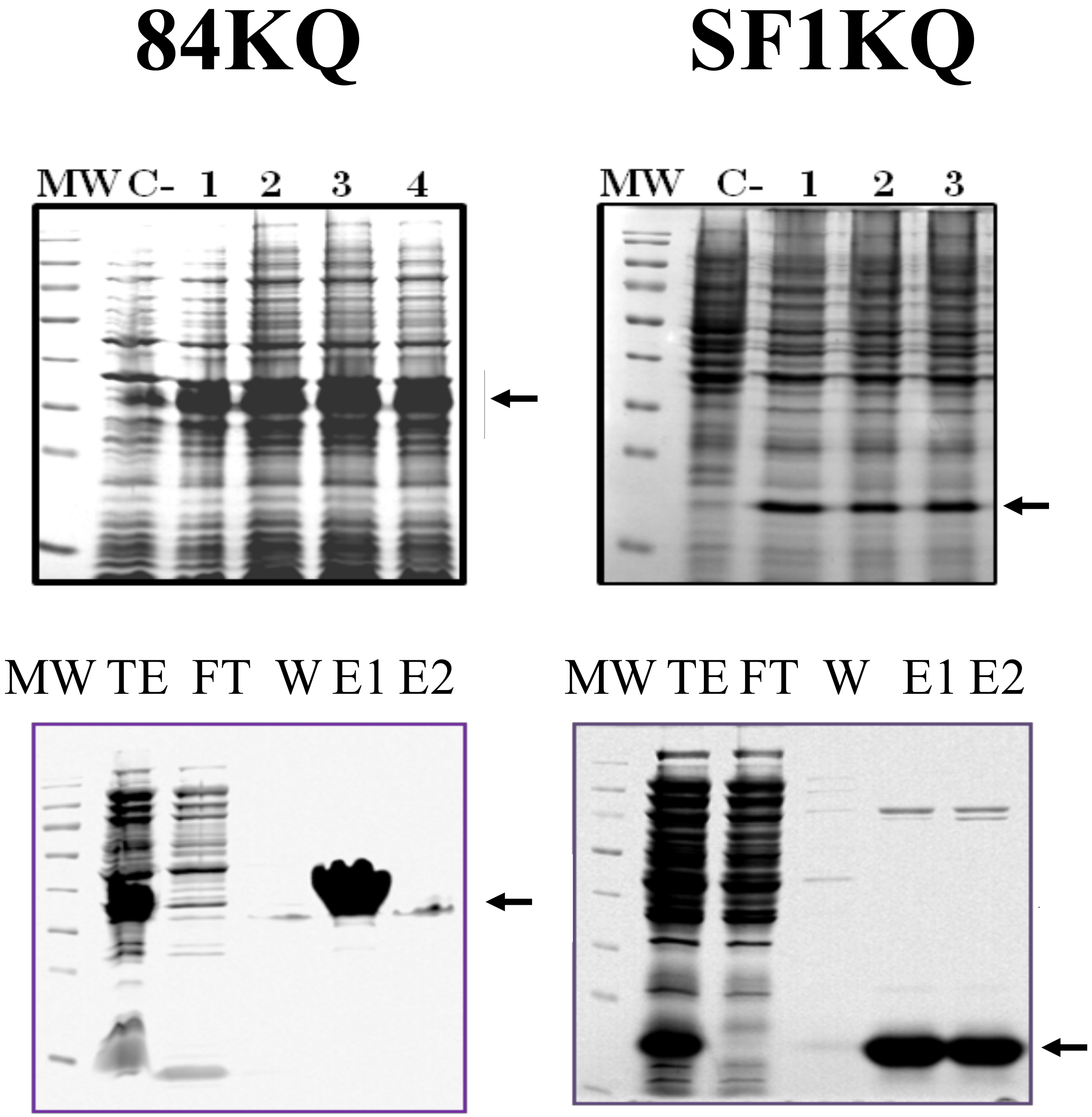

Supplementary Figure 3. Compared to the untransformed strain (C-), protein induction of different transformed strains (numbered lanes) was monitored in Coomassie Blue-stained 12% SDS-PAGE. Corresponding domains are labeled on the top. In the bottom gels, aliquots of the different purification steps (TE, total extract; FT, flow-through; W, wash; and E, elution fractions) were resolved. MW, relative mobility molecular markers. Arrows indicate the induced and purified recombinant proteins.

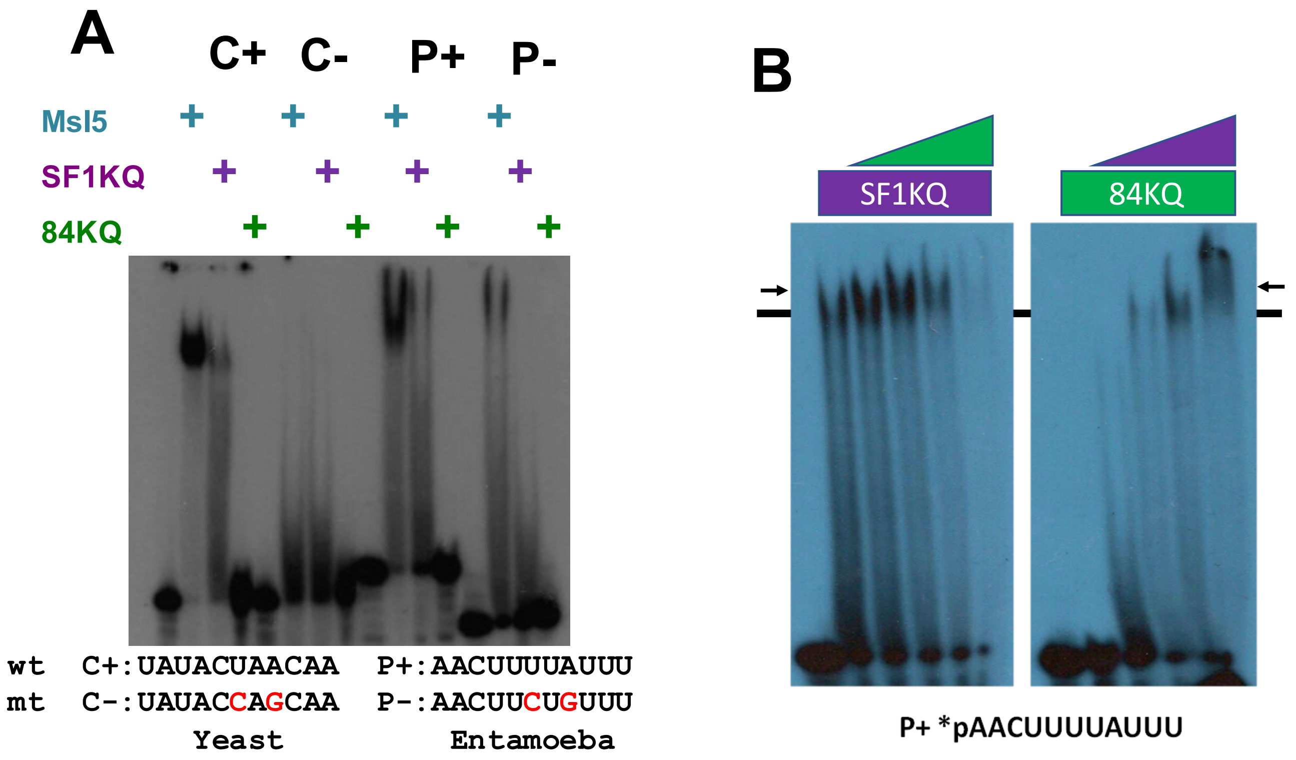

Supplementary Figure 4. The 84KQ domain supershifts the complex formed between the SF1KQ domain and the branchpoint sequence. (**A**) EMSAs were carried out to verify whether the recombinant SF1KQ, 84KQ, and yeast Msl5 (ortholog of SF1) proteins were able to bind to the yeast control (C+: *pUAUACUAACAA) and the RabX13 intron branch point sequence (BS) radioactive RNA probes (P+: *pAACUUUUAUUU), but not to mutant BS probes (C-: *pUAUACcAgCAA; P+: *pAACUUcUgUUU). (**B**) 5 µg of SF1KQ or 84KQ recombinant proteins (purple and green rectangles, respectively) were incubated without or with increasing amounts of the reciprocal protein 84KQ and SF1KQ (0.5, 2.5, 5, and 50 µg; green and purple triangles, respectively). The thick line indicates the SF1KQ-BS complexes and arrows indicate the 84KQ supershifted complexes.

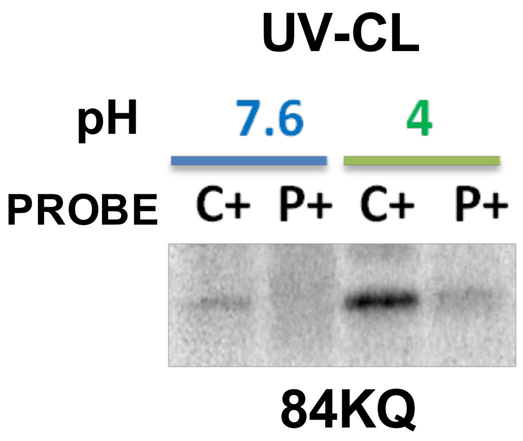

Supplementary Figure 5. 84KQ does recognize branchpoint sequence RNAs at low pH. UV-crosslinking experiments were carried out with C+ and P+ radioactive BS RNA probes and recombinant 84KQ protein (estimated pI 4.02, according to Prot-param) at pH 7.6 (neutral) and pH 4 (acidic). Because the yeast BS probe is consensual (NCURAY) it is better recognized by the recombinant 84KQ protein than the Entamoeba BS probe which deviates from the consensus by two residues (in lower case, UuUuAU).

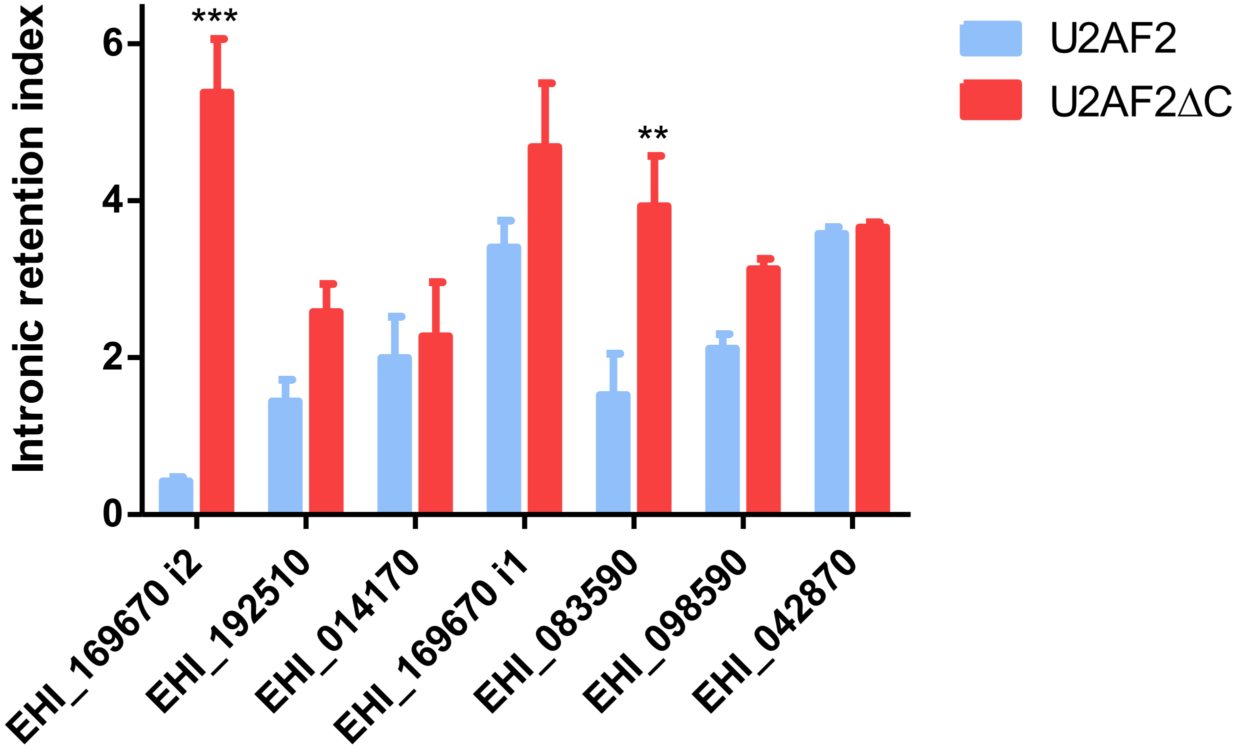

Supplementary Figure 6. The absence of the 84KQ affects the expression of some virulence-related transcripts. Transcription expression (intron retention + mRNA) was obtained from the *in vivo* splicing assays comparing full-length and EhU2AF2EhU2AF2ΔC transformants. Statistical significance (Two Way ANOVA Bonferroni post-test, $**P < 0.01$, $*** P < 0.001$; nonsignificant $P > 0.05$).

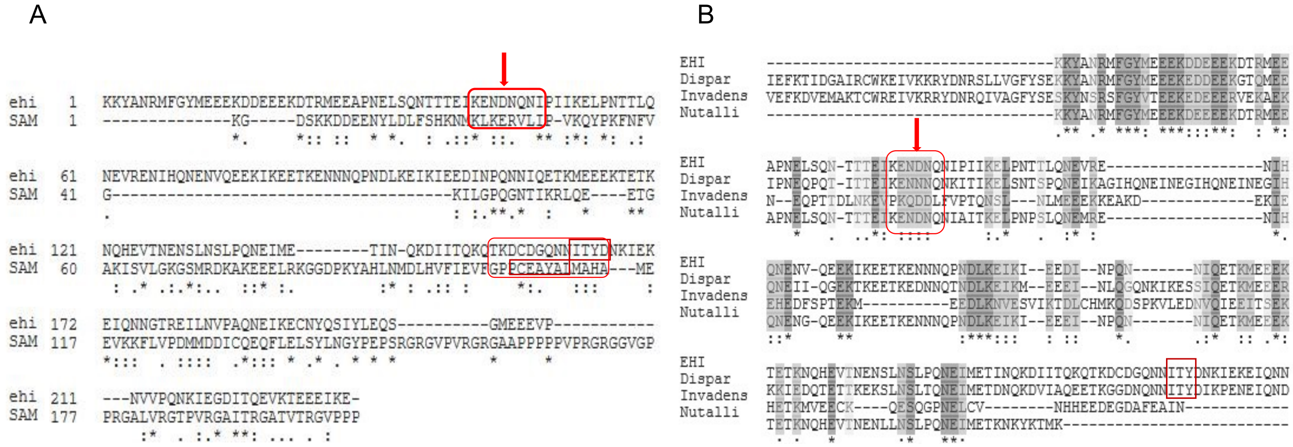

Supplementary Figure 7. Candidate amino acids for EhU2AF2/EhSF1 KH-KH dimerization. Previous works identified the residues pairs Q58 and Y141, and E158 (red arrow) and Y241 (red cartouche) of the T-STAR and Sam68 proteins, respectively (Feracci et al., 2016; Teplova et al., 2013) involved KH-mediated protein dimerization. To identify the putative KH residues involved in splicing factors dimerization, the KH motifs of the proteins T-STAR, Sam68, Gdl1, KQI, and SF1 were aligned against SF1, and the KH-QUA2 domain of U2AF2 from *E. histolytica*, *E. dispar*, *E. invadens* and *E. nuttali* using CLUSTAL-W. Examples of the alignments show the human Sam68 compared to *E. histolytica* (**A**) and the comparison of U2AF2 from the four Entamoeba species (**B**). From these alignments, we identified that residues D522 (red arrow) and Y643 (red cartouche) represent the best candidates for KH-KH dimerization.

1.2 Supplementary Tables

Supplementary Table 1. Genes, oligonucleotides, and conditions used in the experiments are described in the main text. Gene accession numbers are in parenthesis. Amplifications were done at 35 and 38 cycles. US, unspliced, and S, spliced variants.

Gene	Oligonucleotides (5' ➔ 3')		PCR conditions	Amplicon length
RabX13 (EHI_065790)	Rab2 F<br>Rab2 R	CGTTGTTGGAGACTCTTCAGTTGG<br>GACCCATTTCAGTTGAAACAGTTC	94°C,45'', 60°C,45'', 72°C,45''	US = 510 bp<br>S = 374 bp
ClcB (EHI_186860)	ChloF<br>ChlRE2	ATGGAACAAAATTTACCCTC<br>CTATTGGAAGTGGATTATGGTG	94°C,45''; 55°C,45''; 72°C,30''	US = 652 bp<br>S = 596 bp
Cdc2 (EHI_065280)	Cdc2 F<br>Cdc2 R	CAATTAGGAGAAGGAACATATGG<br>GTGGTTTCATATCTCTGTGAAG	94°C,45''; 54°C,45''; 72°C,30''	US = 440 bp<br>S = 361 bp
Sam50 (EHI_078220)	FSam50<br>RSam50	GCAATGACAACAAGAATGCAA<br>AAGAAACCCCAACTCCACAA	94°C,45''; 64°C,45''; 72°C,30''	US = 835 bp<br>S = 763 bp
U2AF84 (EHI_098300)	Fstu<br>Rsac	AAAAGGCCTATGGCAGGAAGGTATG<br>CCATGAGCTCTTAATTGTTGAGATATTT	94°C,15''; 60°C,30''; 72°C,1'	2139 pb
EHI_169670	Pr-169670I1 ExU F<br>Pr-169670I1 ExU R	AAGATAAAGAAATAGAAGATGCAGTTAAAG<br>CTTCAGCAGCAACTGAAATTG	95°C,50''; 52°C,45''; 72°C,50''. Melt 52°C,0.5''	132 pb
	Pr-169670I1 IR F<br>Pr-169670I1 IR R	GAAGATGCAGTTAAAGAAAAAGA<br>CTTCAGCAGCAACTGAAATTG	94°C,45''; 52°C,45''; 72°C,45''	118 pb
	Pr-169670I2 ExU F<br>Pr-169670I2 ExU R	TTAAAAAAGAAAAAGAAGTTTTGTCTA<br>GATGTTTCAAATCTTCTTTCTCCTTC	95°C,50''; 52°C,45''; 72°C,50''. Melt 52°C,0.5''	126 pb
	Pr-169670I2 IR F<br>Pr-169670I2 IR R	GAAGTTTTGTCTAAAGAAAACAAAC<br>GATGTTTCAAATCTTCTTTCTCCTTC	94°C,45''; 52°C,45''; 72°C,45''	112 pb
EHI_192510	Pr-192510 ExU F<br>Pr-192510 ExU R	TATATCAAGATGGGAAAAACTACAAAGATA<br>TGGCTTTGTCACTGTTGCTT	95°C,50''; 52°C,45''; 72°C,50''. Melt 52°C,0.5''	178 pb
	Pr-192510 IR F<br>Pr-192510 IR R	AACTACAAAGATAAAATTGATTAT<br>TGGCTTTGTCACTGTTGCTT	94°C,45''; 52°C,45''; 72°C,45''	120 pb
EHI_014170	Pr-014170 ExU F<br>Pr-014170 ExU R	TAACTCAACAAAAGAAAATTTCTTCTTTAG<br>TTTCTTTCTTTAATTCTTCAAGT	95°C,50''; 50°C,45''; 72°C,50''. Melt 50°C,0.5''	186 pb
	Pr-014170 IR F<br>Pr-014170 IR R	CATTAAAAATTTCTTTAGCAAAT<br>TTTCTTTCTTTAATTCTTCAAGT	94°C,45''; 54°C,45''; 72°C,45''	172 bp
EHI_042870	Pr-042870 ExU F<br>Pr-042870 ExU R	ATTGAATATATTGATAGACTATTTCAAAGCC<br>TCACATCCACATACTTCTTCTGG	95°C,50''; 50°C,45''; 72°C,50''. Melt 50°C,0.5''	197 pb
	Pr-042870 IR F<br>Pr-042870 IR R	AGACTATTTCAAAGCCTCAACCA<br>TCACATCCACATACTTCTTCTGG	94°C,45''; 49°C,45''; 72°C,45''	182pb
EHI_083590	Pr-083590 ExU F<br>Pr-083590 ExU R	TTGGAGGTAAAGGAGAAAAACCACAATC<br>GGTACACAGAAAGAGACTGGAACA	95°C,50''; 50°C,45''; 72°C,50''. Melt 50°C,0.5''	126 pb
	Pr-083590 IR F<br>Pr-083590 IR R	ACAATCTGGATTAGGAGTTATT<br>GGTACACAGAAAGAGACTGGAACA	94°C,45''; 55°C,45''; 72°C,45''	114 pb
EhRNF (EHI_098590)	EHI_098590 ExU F<br>EHI_098590 ExU R	CCAGACTTTTTGATGTTAGAACAAAAACG<br>TTGGAGGTGGGGTATATCGT	95°C,50''; 50°C,45''; 72°C,50''. Melt 50°C,0.5''	121 bp
	EHI_098590 IR F<br>EHI_098590 IR R	CCAGACTTTTTGATGTTAGAACAAAAACG<br>TTGGAGGTGGGGTATATCGT	94°C,45''; 55°C,45''; 72°C,45''	106 bp
EhRNA polymerase II (EHI_056690)	RNAPIIs<br>RNAPIIas	GATCCAACATATCCTAAAACAACA<br>TCAATTATTTTCTGACCCGTCTTC	94°C,45'', 60 °C,45'', 72 °C,45'	204 bp
EhU2AF2 (EHI_098300)	U2AF84Cf s<br>Eh84Nhe as	CACACCCGGGATGGCGTATGATAGGTCTCG<br>ATCTGAGGCTAGCGTATCCAAACATTCTATTTGC	94°C,45'', 60°C, 45'', 72°C,1'	1468 pb
EdU2AF2(EDI_161905)	EdNheF s<br>XhoEd84 as	ATCTGAGGCTAGCATGGAAGAAAAAGA AGATG<br>AATCTCGAGTCATTCTTTTCTTTC	94°C,45'',60°C,1', 72°C/1'	825 pb
Eh::dU2AF2	U2AF84Cf s<br>XhoEd84 as	CACACCCGGGATGGCGTATGATAGGTCTCG<br>AATCTCGAGTCATTCTTTTCTTTC	94°C, 45'',55°C, 1',72°C, 2:30'	2293 pb

Supplementary Table 2. The sequences of *Entamoeba histolytica* and *E. dispar* splicing factors show the ULM (red), UHM (blue), and KH-QUA2 (yellow) motifs.

**EhU2AF2**<br>EHI_098300	MAGRYDRSRSRERRYDRDSRSVSRRRRSDYRSSDYSRERDDRRRYYDRYDRREYSPRDRIRYKSS<br>RRRTRREYSRNEDREDRHRRVPEEERYNRSIRRRADRSPSLSPLGDKLPSRWDEQPKAIDSVQISQQ<br>LNVHQERAAKRIYVGNINSSTSEKDIVDAFNEAMRRGDYVDKNDTRDIITHIEVNYERSYAFLEFRTLEE<br>AVKALSLDGLTIKGASVKVRRPKDYNPVLPFISGLSQLMEPGTTNPRESILYMGNIPLQMTDEQIRKKLE<br>NLNPLKNFFVIRDPDLGAPQGKCYCLFEYQNPEYKEKILTFDGINLGGNKIEVCSGVDGFKHLPKASLN<br>ELFSKMFPHTTDLVIGTLLNSSVGYSTVFEKILKPSEKIEDQHVSRIIIIFNMVYPEDLTDQQRYIELIDDIR<br>FVCQEYGEVESISIPRPTEENKKPSGLGRVFIEFKTIEGAIKCWKEIIKKRYDNRSLLVGFYSEKKYANR<br>MFGYMEEEKDDEEEKDTRMEEAPNELSQNTTTEIKENDNQNIPIIKELPNTTLQNEVRENIHQNENVQE<br>EKIKEETKENNNQPNDLKEIKIEEDINPQNNIQETKMEEEKTETKNQHEVTNENSLNSLPQNEIMETINQ<br>KDIITQKQTKDCDGQNNITYDNKIEKEIQNNGTREILNVPAQNEIKECNYQSIYLEQSGMEEEVPNVVPQ<br>NKIEGDITQEVKTEEEIKE
**EhU2AF1**<br>EHI_183100	MERDRFSSRTNERRERYNDRFERRERSYDDYNSHERRREERYEYRNRHEKREEKKGRKEEEVNDK<br>ESIMQQIEKERRTVFVRGLTTEASNEEIKEFFNQAGEVVHVEQVIDTTTKRSRGFGYVEFKTIEGAMKAI<br>EMSGMFFKESSPIYVSESNAQQNRNTITISNTQLQTNRIKVKNMNKQLSKEDIEKVFGIAGKIIELSIQEE<br>EESNNVIIEYDTIKMAKRAIELYDGRSFGNMKWEVFSICEKGSDVITNEDEQLLESKSKEMMLKRIQGG<br>VGTLFGQENINHYKALFVQNVFTQGKEPIGFEKELRNDILEELKQYCQVKDVIVDLIHPKGVVFVLCETE<br>VDAKKAFSVMHLRWFNMHLLRVEYYPESKVPIVHQD
**EhSF1**<br>EHI_193510	MEEVKEVDYKERFTHPGGLPIHEKRVSRWDRKIRDVLPNIPVHIPPSLDEKYWDTLAIRIRYEELQYAL<br>GTHRLGLNTERDRTPSPPKQYNENQQEITREMRREEKLKNERLYVVDRAIEIYPSFRIPAELAKPSGKR<br>TKKIYFPKDRPDTNFIGLIIGPRGDNQKRLEKDSGAKISIRGKDPKKLGKLSGYGDKDNEDSHVFITADT<br>QEALDLACEEITKIISAPSEEINVLKHNQLRELALWNGTFREDRVYEVEQYESGVKCGFCGDSSHATCD<br>CPLKKQKMSEHQLELERAFDEFMEKIQLQLIN
**EhSF3B1**<br>EHI_049170	MSNSSESESRHRRRSESYSSDYSPRPRRSRWDEKPTVVSTLIDTNEKPDATPALISNDIGIQNKIESLP<br>DVKPGERAFFEDALIQRDESELSSEERKKIQLKRLLLRIKNGTPAMRKQALRQLTERTKEFGAEVLFEQI<br>LPLLMSITLEQQERHILVKVVNRIIFKLDSLVRPFTAKLLVVITPLLDDADFIARVEGREIISNLAKAAGLQT<br>MIAAMRPDIDSPEESIRNTTARAFSVVAAAIGIPSLLPFLKAVCGSKKSWYARHTGLKCIQQIAILMGCSV<br>LPHLSALVAIVFPRLNDIEANITKFAALAIAALAEASYPYGGDVLEQTLEPIIEGCKRMRGRLLASYIKAAG<br>QVISVVDEEIAAKYGWEIIRVVVREFKTSDEEMKKIVLKVIRQCLNVEIIGKETSKNNIAERFFESFWHRR<br>NSVDKKLSKEVIETALLLSQKMGAKYILEKIVVFLKDENEPFRKMTLKAMEKVIQQFGIYEIDEDLEKRIF<br>DGLTFAFIEQTSGDENAQALQSISFIMNCFSERVVPYLEAFSENIKWRFHNKSPKIRQCAVEILGNICGL<br>YIKCNQKPTLIDLCEILYELLGESNTEVLASTMITLKEIISLCNLEEIRPSISDLVPGLTPILRNTNERIEEACI<br>GLIGIIAKKSADTGAEMVHLKEWMRICHELLDAFKAHKKSIRRATVDTFGDIAKAIGPQEVLIMLLNNLKV<br>LDRQLRVCTTIAIAVVADSCAPFTVIPSLMNEYRMPDINIKTGVLKAFAFLFEYIGEKSKDYIYPVIPLLCD<br>ALAEKDAVHRQTACTVVKFISLGIYGLGCEDALIHMLNYVWPNIFETSPHVINATLEALEGIRVSLGVLVL<br>MQYVLQGLFHPARHVREPYWRVYNNMYIGNQDGLVAAYPVLEDDEYNMNRRYELEILL
**EhTIA-1**<br>EHI_056660	MTRQYDSRYQYPPRAPQLYQQRPPQQSSTETTESSLPINANSKSVHVSGIHESVDEILLGRIFSIVGHV<br>VSCKIMRDKSGVHAGYGFVEFVDSTTARFAKDNMDGRVVYGRELKVNWSYTAQQENQGNYKIFVGG<br>LQPEVNDDLLYKTFQKFGRVTDARVLKFTQTGKSKGYGFVTFIRKEDAETAMQMMNGEKLEGRNIKV<br>NWVTSNIASKTEQPKRSYDEINNETSSQNCTVYIGNIPKNVESDDLKQLLAEYGSIEEVRLNKDKGYAFI<br>KFSKHESATSAILMCNGKIINGSTLRCSWGRESH
**EdU2AF2**<br>EDI_161905	MAGRYDRSRSRERRYDRDSRSVSRRRRSDYRSSDYSRERDDRRRYYDRYDRREYSPRDRIRYKSS<br>RRRTRREYSRNEDREDRHRRVPEEERYNRSIRRRADRSPSLSPLGDKLPSRWDEQPKAIDSVQISQQ<br>LNVHQERAAKRIYVGNINSSTSEKDIVDAFNEAMRRGDYVDKNDTRDIITHIEVNYERSYAFLEFRTLEE<br>AVKALSLDGLTIKGASVKVRRPKDYNPVLPFISGLSQLMEPGTTNPRESILYMGNIPLQMTDEQIRKKLE<br>NLNPLKNFFVIRDPDLGAPQGKCYCLFEYQNPEYKEKILTFDGINLGGNKIEVCSGVDGFKHLPKASLN<br>ELFSKMFPHTTDLVIGTLLNSSVGYSTVFEKILKPSEKIEDQHVSRIIIIFNMVYPEDLTDQQRYIELIDDIR<br>FVCQEYGEVESISIPRPTEENKKPSGLGRVFIEFKTIEGAIKCWKEIIKKRYDNRSLLVGFYSEKKYANR<br>MFGYMEEEKEDDEEKGTQMEEIPNEQPQTITTEIKENNNQNKITIKELSNTSPQNEIKAGIHQNEINEGI<br>HQNEINEGIHQNEIIQGEKTKEETKEDNNQTNDLKEIKMEEEINLQGQNKIKESSIQETKMEEERKKIED<br>QTETTKEKSLNSLTQNEIMETDNQKDVIAQEETKGGDNQNNITYDIKPENEIQNDEIKEVLNISAQNEIKE<br>YDNKSVELGQSKIEEIKENSDTKNFLKKEEEVVPNTSTQNKVEESIIQEVKMEEEKTERKE
